# Extremely Longitudinally Extensive Transverse Myelitis in a Patient With Myelin Oligodendrocyte Glycoprotein Antibody-Associated Disease

**DOI:** 10.7759/cureus.59938

**Published:** 2024-05-08

**Authors:** So Okubo, Toshiyuki Kakumoto, Masahiko Tsujita, Kyosuke Muramatsu, Sho Fujiwara, Masashi Hamada, Wataru Satake, Tatsushi Toda

**Affiliations:** 1 Neurology, University of Tokyo Graduate School of Medicine, Tokyo, JPN

**Keywords:** spinal cord diseases, neuro-immunology, mog associated antibody disease (mogad), anti-mog antibody, longitudinally extensive transverse myelitis

## Abstract

Longitudinally extensive myelitis with 15 or more vertebrae in length is extremely rare, with limited evidence regarding clinical features and therapeutic response. We report a case of a 29-year-old male patient with extremely longitudinally extensive myelitis ultimately diagnosed as myelin oligodendrocyte glycoprotein-associated disease (MOGAD). The patient presented with an acute onset of meningismus, limb weakness, sensory disturbance below the C5 level, ataxia, and urinary retention. T2-weighted imaging on MRI showed an extremely longitudinally extensive spinal cord lesion ranging from C2 to the medullary conus, together with a left pontine lesion. Positive anti-myelin oligodendrocyte glycoprotein antibodies were serologically detected, which led to the diagnosis of MOGAD. Intravenous methylprednisolone followed by 1 mg/kg oral prednisolone with taper resulted in complete symptomatic and radiological resolution. The striking complete resolution despite the symptomatic and radiological severity observed in this case has been described in a few previously reported MOGAD cases. Extremely longitudinally extensive myelitis with excellent therapeutic response may be a characteristic presentation of MOGAD.

## Introduction

Longitudinally extensive transverse myelitis (LETM) is defined as a spinal cord lesion involving three or more vertebral segments [1]. LETM has classically been associated with neuromyelitis optica spectrum disorder (NMOSD); however, the etiology of LETM includes a wide variety of inflammatory and non-inflammatory neurological diseases [2]. Extremely longitudinally extensive transverse myelitis (ELETM) spanning more than 15 vertebral segments are a rare subset of LETM with limited reports on etiology and therapeutic response. Here, we present a case of ELETM ultimately diagnosed as myelin oligodendrocyte glycoprotein-associated disease (MOGAD).

## Case presentation

A 29-year-old male patient visited our institution with an acute onset of fever and headache lasting 12 days. No other systemic symptoms of febrile illness were present. Six days before admission, weakness in the lower extremities, sensory disturbance in both feet, and dysuria emerged and rapidly progressed. On the day of admission, the patient was no longer able to walk. 

**Figure 1:**
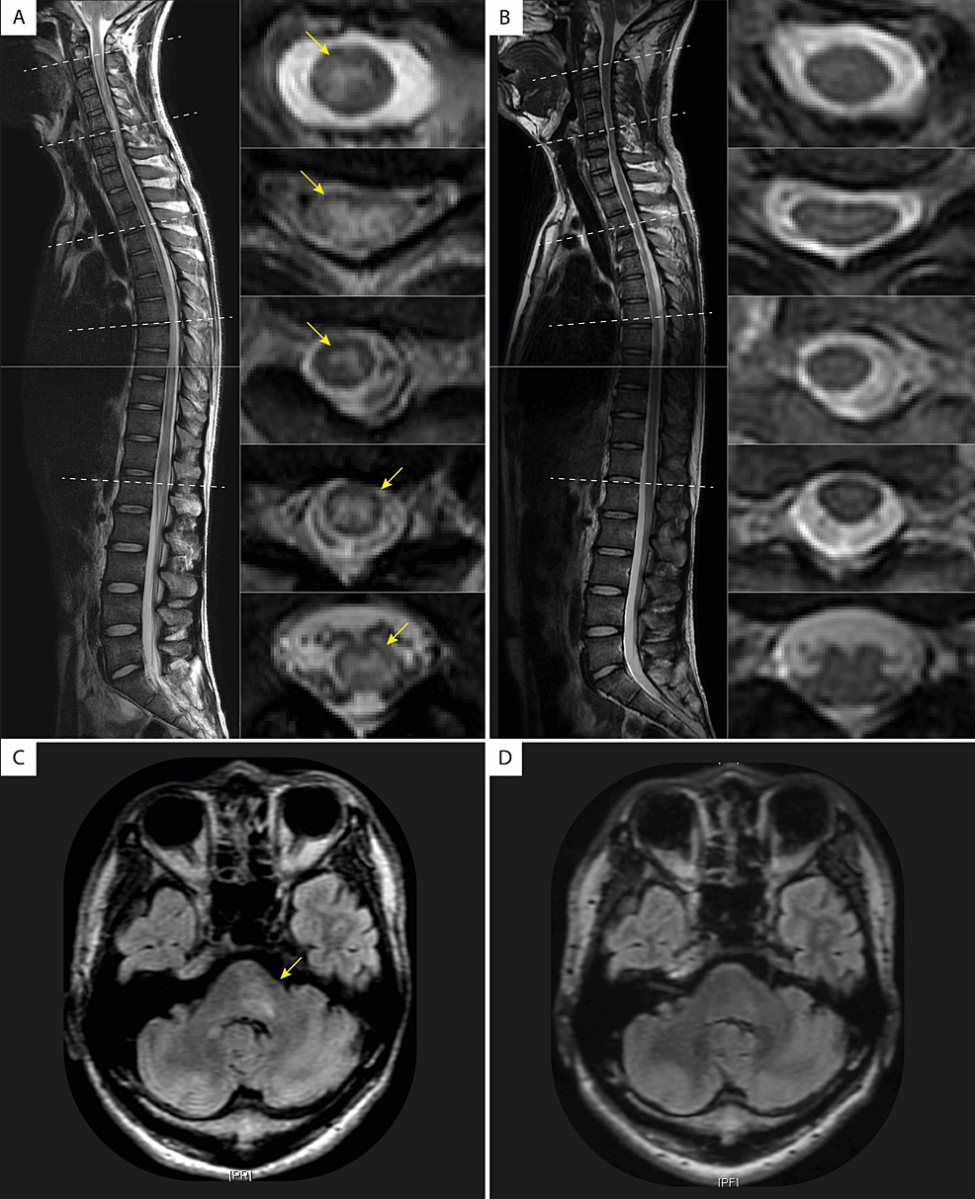
Radiological findings of the patient before and after treatment. A) Saggital and axial views of T2-weighted MRI of the spinal cord before treatment showed a high-intensity lesion ranging from C2 to the conus. B) Saggital and axial views of T2-weighted MRI 28 days after admission showed complete resolution of high-intensity lesions. C) Fluid attenuated inversion recovery (FLAIR) sequence of the axial view of the brain MRI before treatment showed a high-intensity lesion in the left side of the pons (arrowhead). D) The left pontine lesion was not evident in the FLAIR sequence of the axial view of the brain MRI after treatment.

Physical examination on admission revealed fever (38.0 ℃) and signs of meningeal irritation. Neurological examination revealed diffuse limb weakness. The Medical Research Council (MRC) scale for muscle strength was 4 for the distal upper extremities and proximal lower extremities and 2 for the distal lower extremities. Tendon reflexes were exaggerated in the right upper extremity and absent in the lower extremities. Sensory disturbance was evident below the C5 level. Left-sided ataxia and urinary retention were also noted. The patient could not stand. The expanded disability severity scale (EDSS) was rated as 7.0. Cerebrospinal fluid (CSF) analysis showed elevated levels of protein (102 mg/dL) and elevated white blood cell count (211 cells/μL). CSF-restricted oligoclonal bands were not detected, and the IgG index was not significantly elevated (0.66). Infectious pathogens in the CSF were tested negative by the FilmArray® meningitis/encephalitis panel (BioMérieux, Lyon, France), a multiplex PCR-based microbial detection system targeting bacteria (*Escherichia coli* K1, *Haemophilus influenzae*, *Listeria monocytogenes*, *Neisseria meningitidis*, *Streptococcus agalactiae*, and *Streptococcus pneumoniae*), viruses (Cytomegalovirus, Enterovirus, Herpes simplex virus 1, Herpes simplex virus 2, Human herpesvirus 6, Human parechovirus, and Varicella zoster virus), and cryptococcus (*Cryptococcus neoformans* and *Cryptococcus gattii*). T2-weighted imaging on magnetic resonance imaging (MRI) showed an extremely longitudinally extensive spinal cord lesion ranging from C2 to the medullary conus (Figure 1A), along with a left pontine lesion (Figure 1C). Contrast enhancement was not evident in these lesions. Anti-aquaporin 4 (AQP4) antibody testing of the serum by live cell-based assay (CBA) was negative. Positive anti-myelin oligodendrocyte glycoprotein (MOG) antibodies were detected serologically by live CBA (titers 1:64, normal limit <1:16), which led to the diagnosis of MOGAD. Two cycles of intravenous methylprednisolone (1 g/day for three consecutive days per cycle, one-week interval between cycles) followed by oral prednisolone 1 mg/kg with taper resulted in complete symptomatic and radiological resolution (Figures 1B, 1D). On day 54 of admission, the patient was discharged from the hospital free of neurological symptoms (EDSS 0.0). The patient has not experienced signs of relapse as of five months follow-up after discharge.

## Discussion

Longitudinally extensive myelitis with 15 or more vertebrae in length is extremely rare, with limited evidence regarding clinical features and therapeutic response. Of the four cases previously reported, one was positive for anti-AQP4 antibody and three were positive for anti-MOG antibody [3-6]. Myelitis involving C1 to the conus in one patient with positive anti-AQP4 antibody only responded partially to treatment [3]. Of the three MOGAD patients, information on therapeutic response was available in two patients. The two patients displayed substantial disability at onset and were unable to walk without aid. Similar to our case, both of these patients showed symptomatic and radiological resolution after steroid administration, contrasting the AQP4-antibody positive case [4,5]. In a previous study on transverse myelitis, which included both short and long lesions, overall mobility recovery was better in MOGAD patients compared to AQP4-antibody positive cases [7]. Our findings together with the previously reported cases suggest a favorable therapeutic response in MOG-associated ELETM. It is of note that disease-modifying therapies against NMOSD targeting complement factor C5, B-lymphocytes, and interleukin-6 have emerged recently, showing a reduction in relapse risks [8-11]. Considering the substantial disability risk associated with each acute attack [12], the decreased relapse risk with these therapies may have improved the prognosis of anti-AQP4 antibody-positive LETM compared to previous reports. 

This case illustrates the striking complete resolution despite the symptomatic and radiological severity observed in MOG-associated ELETM. The effect of lesion length on prognosis has been implicated in a multicenter study comparing MOGAD patients with short myelitis versus LETM [13]. A worse prognosis on the basis of EDSS was observed in the LETM group compared to the short myelitis group. Furthermore, a high EDSS at onset was associated with a poor prognosis. The difference in therapeutic response between MOG-associated ELETM, including this study, and MOG-associated LETM with intermediate length may be attributable to other prognostic factors, including age [14] or possibly early intervention. Because the ELETM cases were scarce, further studies are necessary to uncover the therapeutic response of ELETM. 

## Conclusions

ELETM spanning more than 15 vertebral segments is rare, and reports on therapeutic response are still limited. Extremely longitudinally extensive myelitis with excellent therapeutic response may be a characteristic presentation of MOGAD. Early initiation of acute attack treatment may be key for this excellent response. Further investigation to determine the clinical characteristics of extremely longitudinally extensive myelitis may allow for better-targeted therapeutic strategies.
